# Global Analysis of Viral Infection in an Archaeal Model System

**DOI:** 10.3389/fmicb.2012.00411

**Published:** 2012-12-10

**Authors:** Walid S. Maaty, Joseph D. Steffens, Joshua Heinemann, Alice C. Ortmann, Benjamin D. Reeves, Swapan K. Biswas, Edward A. Dratz, Paul A. Grieco, Mark J. Young, Brian Bothner

**Affiliations:** ^1^Department of Chemistry and Biochemistry, Montana State UniversityBozeman, MT, USA; ^2^Department of Marine Science, University of South AlabamaMobile, AL, USA; ^3^Department of Microbiology, Montana State UniversityBozeman, MT, USA; ^4^Department of Plant Sciences, Montana State UniversityBozeman, MT, USA

**Keywords:** archaea, virus infection, *S. solfataricus P2*, proteomics, virus–host interaction, differential gene expression, activity probes, caspase

## Abstract

The origin and evolutionary relationship of viruses is poorly understood. This makes archaeal virus-host systems of particular interest because the hosts generally root near the base of phylogenetic trees, while some of the viruses have clear structural similarities to those that infect prokaryotic and eukaryotic cells. Despite the advantageous position for use in evolutionary studies, little is known about archaeal viruses or how they interact with their hosts, compared to viruses of bacteria and eukaryotes. In addition, many archaeal viruses have been isolated from extreme environments and present a unique opportunity for elucidating factors that are important for existence at the extremes. In this article we focus on virus-host interactions using a proteomics approach to study *Sulfolobus* Turreted Icosahedral Virus (STIV) infection of *Sulfolobus solfataricus P2*. Using cultures grown from the ATCC cell stock, a single cycle of STIV infection was sampled six times over a 72 h period. More than 700 proteins were identified throughout the course of the experiments. Seventy one host proteins were found to change their concentration by nearly twofold (*p* < 0.05) with 40 becoming more abundant and 31 less abundant. The modulated proteins represent 30 different cell pathways and 14 clusters of orthologous groups. 2D gel analysis showed that changes in post-translational modifications were a common feature of the affected proteins. The results from these studies showed that the prokaryotic antiviral adaptive immune system CRISPR-associated proteins (CAS proteins) were regulated in response to the virus infection. It was found that regulated proteins come from mRNAs with a shorter than average half-life. In addition, activity-based protein profiling (ABPP) profiling on 2D-gels showed caspase, hydrolase, and tyrosine phosphatase enzyme activity labeling at the protein isoform level. Together, this data provides a more detailed global view of archaeal cellular responses to viral infection, demonstrates the power of quantitative two-dimensional differential gel electrophoresis and ABPP using 2D gel compatible fluorescent dyes.

## Introduction

Identifying and understanding the interplay of viral and host factors during cell entry, replication, and egress is critical to deciphering the events that determine the fate of infection. Such studies have historically played an important role in elucidating fundamental aspects of molecular and cellular mechanisms. Archaeal host-virus interactions are just beginning to be explored and the current understanding of archaeal virus mechanisms is rudimentary at best. This is especially true of viruses that infect members of the crenarchaea. Even for the first viruses described that infect the crenarachaeal host *Sulfolobus* species, such as *Sulfolobus* spindle-shaped virus (SSV; Wiedenheft et al., 2004) and *Sulfolobus islandicus* rod-shaped virus (SIRV; Kessler et al., 2004), the viral replication cycle is just beginning to be understood. The recently described *Sulfolobus* Turreted Icosahedral Virus (STIV; Rice et al., 2004; Maaty et al., 2006) has emerged as a model crenarchaeal virus system due to the availability of a sequenced genome (Rice et al., 2004), infectious clones which facilitate genetic manipulations (Wirth et al., 2011), and detailed structural information on the STIV virion (Larson et al., 2006, 2007a; Khayat et al., 2010) and many of the structural and non-structural components (Maaty et al., 2006). Major findings include an icosahedral virion architecture with an internal lipid membrane, turret structures on the surface, and the discovery that STIV has evolved a novel release mechanism that involves the creation of pyramidal structures on the surface of infected cells (Brumfield et al., 2009; Snyder et al., 2011; Fu and Johnson, 2012). Most strikingly, there appears to be a evolutionary relationship at the structural level with prokaryotic and eukaryotic viruses (Khayat et al., 2005; Maaty et al., 2006). STIV has arguably become one of the most studied crenarchaeal archaeal virus systems.

Crenarchaeal viruses form a distinct yet highly diverse group. The description of less than 50 viruses has led to at least seven new families (Globuloviridae, Guttaviridae, Fuselloviridae, Bicaudaviridae, Ampullaviridae, Rudiviridae, and Lipothrixviridae) with viruses such as STIV still awaiting assignment (Lawrence et al., 2009). All have circular double-stranded DNA genomes except for the Rudiviridae and Lipothrixviridae which are the only known viruses to have linear dsDNA. To date, there is no description of ssDNA or ssRNA archaeal viruses, however, recent evidence from metagenomic analysis of archaeal dominated hot springs in Yellowstone National Park (YNP) identified novel positive-strand RNA viruses (Bolduc et al., 2012). While most archaea possess CRISPR/Cas antiviral systems, the mechanism of this or other viral counter measures have yet to be worked out. Originally, a number of the crenarchaeal viruses, including STIV and SIRV2, were not believed to cause cell lysis (Prangishvili and Garrett, 2005). It was later revealed that both of these viruses have very narrow host ranges and are only able to infect a sub-population of cells in a stock culture (Ortmann et al., 2008; Quax et al., 2011). The ability of STIV and SIRV2 to cause cell lysis was clearly demonstrated when both were shown to produce viral associated pyramids (VAPs) on the surface of host cells that opened to release mature particles (Brumfield et al., 2009; Prangishvili and Quax, 2011). Previously only eukaryotic viruses were known to produce replication structures and nothing like the VAPs had ever been described before.

*Sulfolobus* Turreted Icosahedral Virus was isolated from enrichment cultures of a high temperature (∼80°C) acidic (∼pH 2.9–3.9) hot spring in YNP (Rice et al., 2001). This was the first icosahedral virus described with an archaeal host. It has been shown to infect *S. solfataricus* (P2), originally isolated from Italy, as well as several *Sulfolobus* species found in YNP. Structural models based on cryo-electron microscopy and image reconstruction revealed that the STIV capsid has pseudo *T* = 31 symmetry, turret structures at each of the fivefold axes, and an internal lipid monolayer (Rice et al., 2004; Khayat et al., 2005). Subsequent analysis determined that the capsid is composed of nine viral proteins and an internal cyclic tetraether lipid layer, which is selected from the host lipid compliment (Maaty et al., 2006). The 17.6 kb dsDNA genome has 38 recognized open reading frames (Maaty et al., 2012). Functional and evolutionary insight for the gene products of 4 open reading frames has come from crystal structures of A197, F93, B116, and the major coat protein (Larson et al., 2006, 2007a,b).

Global untargeted analyses of gene and protein expression profiles are powerful approaches for examining viral infection (Der et al., 1998). An advantage of untargeted approaches is the ability to detect novel and unexpected connections that would be missed with a more focused approach. Proteomics approaches including methods such as shot-gun liquid chromatography-mass spectrometry, differential gel electrophoresis (DIGE), and isotope coding facilitate quantitative analyses of whole proteomes. DIGE, which is based on 2-dimensional electrophoresis (2D), allows differences between independent samples labeled with different fluorescent dyes to be detected on a single gel. This differential approach improves reproducibility and facilitates comparison based on statistically significant changes between thousands of proteins in parallel (Tonge et al., 2001; Gharbi et al., 2002; Van den Bergh et al., 2003). Fluorescence-based DIGE is quantitative over a large range of protein concentrations and has an inherent ability to detect post-translational modifications (PTMs) and changes in PTMs based on changes in protein mobility. A limiting factor with the 2D-DIGE approach is that generally only ∼1000 proteins can be separated on a single gel. For complex eukaryotic samples, prefractionation or the use of narrow pH range isoelectric focusing strips may be required. With prokaryotic species such as *Sulfolobus* (∼3,000 genes), a single wide pI range 2D gel provides a high degree of proteome coverage of the expressed proteins.

While measurements of changes in mRNA, protein, and protein PTMs are powerful approaches and are the foundation of functional genomics, they are only proxies for biological activity. Activity-based protein profiling (ABPP) on the other hand, is a direct read-out of protein function. ABPP uses semi-targeted probes to covalently label enzymes in specific catalytic classes by taking advantage of active site chemistry. Probes have been developed for a wide range of enzyme classes. A distinct advantage of ABPP is that changes in enzyme activity provides a direct read-out of biological change, yet can be followed at the level of the proteome (Speers and Cravatt, 2004; Sadaghiani et al., 2007). This approach overcomes the problem that abundance of mRNA or protein frequently does not directly correlate with protein activity. The application of ABPP to cancer and viral infection has already led to significant findings (Furman et al., 2009; Wang et al., 2009; Blais et al., 2010; Nomura et al., 2010; Singaravelu et al., 2010). In this study we used three new types of aqueous soluble rhodamine dyes derivatized with different activity-based probes to extend ABPP to use with 2D-DIGE.

The goal of this study was to expand what is known about host-virus interactions in archaea using untargeted 2D-DIGE and ABPP. Specifically that study was interested in how cultures of *S. solfataricus* (P2; ATCC) stock cells respond to virus. Based on cell counts of infected/non-infected cells, it was previously reported that 10% of cells in ATCC stock cultures support STIV replication (Ortmann et al., 2008). In contrast to a recent report that followed the *S. solfataricus* P2-2-12 substrain that is readily infected by STIV (∼100% of the cells) and showed a rather weak host response in the proteome (Maaty et al., 2012), the *S. solfataricus P2* cultures had a strong proteomic response to virus. *S. solfataricus* P2-2-12 is a susceptible strain isolated by single colony plating (Ortmann et al., 2008). The analysis followed a time course of STIV inoculated *S. solfataricus P2* cultures. Because only a small percentage of cells appear to become infected in such cultures, it was expected that changes at the proteome level would be minimal. Surprisingly, a relatively strong change in protein abundance occurred across the cell population with 40 increasing and 31 decreasing in abundance over the course of 72 h. More than half of these proteins were assigned to just five [clusters of orthologous groups (COG)]. In addition to simple changes in protein concentration, numerous examples of host proteins that were altered post-translationally were detected. This represents the first wide-scale application of activity probes in Archaea and ABPP of serine hydrolases, tyrosine phosphatases, and caspase-like active sites revealed the responsiveness of archaeal enzymes to the probes. A comparison of the regulated proteins to their mRNA half-life revealed that proteins showing changes in abundance came from mRNAs with shorter than average half-lives. *S. solfataricus* (P2) cells clearly respond to STIV, this can be seen across the proteome and includes changes in CAS proteins believed to be part of a CRISPR antiviral system, without significant change to growth rate or morphology.

## Materials and Methods

### Virus purification and infection

Production of the virus was carried out by growing the *S. solfataricus* (P2) cells in Medium 182 (http://www.dsmz.de/microorganisms/medium/pdf/DSMZ_Medium182.pdf) at pH 2.5 as previously described (Ortmann et al., 2008). Briefly, infected cultures were monitored for virus production using an immuno-dot blot assay for the major capsid protein. Cultures were harvested between 48 and 60 h post-infection (hpi). Cells were removed by centrifugation (4000 × *g* for 10 min), and the supernatant containing the virus was filtered through a 0.22-μm Seritop filter (Millipore). The filtrate was concentrated over Amicon membranes (Millipore) with a molecular mass cutoff of 100 kDa until the retained volume was ∼15 mL. For this study, three independent 1 L cultures of *S. solfataricus* (P2) were inoculated with 0.1 mL concentrated STIV (10^11^ virus mL). Preliminary experiments were conducted to determine the amount of virus required to achieve maximal infection and addition of larger volumes of virus did not change culture growth curves or result in greater virus production. Infected cells from 100 mL were recovered at 12 h intervals post-infection (12, 24, 36, 48, 60, and 72 hpi). Uninfected control cultures were grown and sampled in parallel. Epifluorescence performed as previously described (Ortmann and Suttle, 2009).

### 2-D DIGE sample preparation

Sample extracts were prepared from cell suspensions washed with PBS. Pelleted cells were broken by freeze-thaw then mixed in lysis buffer (30 mM Tris-HCl pH 8.5 (4°C), 7 M urea, 2 M thiourea, 4% CHAPS, 50 mM DTT, 0.5% IPG carrier ampholytes and a cocktail of protease inhibitors) for 1 h and the supernatant was clarified by centrifugation. Proteins were purified and concentrated by precipitation with a fivefold volume of cooled acetone, and re-suspended for 1 h. in lysis buffer without DTT. Protein concentration was measured with the RC/DC Protein Assay Kit (Bio-Rad). Samples were kept frozen until use. *S. solfataricus* (P2) protein samples were prepared and labeled with CyDyes according to the manufacturer’s protocol. Briefly, 50 μg of each protein extract was labeled separately at 0°C in the dark for 30 min with 400 pM of the *N*-hydroxysuccinimide esters of cyanine dyes (Cy3 and Cy5 CyDyes; GE Healthcare) dissolved in 99.8% DMF (Sigma). The internal standard, an equimolecular mixture of all the protein extracts, was labeled with Cy2. Labeling reactions were quenched by the addition of 1 μL of a 10-mM l-lysine solution (Sigma) and left on ice for 10 min. Cy2, Cy3, and Cy5 for appropriate control or infected samples were combined and mixed with rehydration buffer (7 M urea, 2 M thiourea, 4% CHAPS) containing 50 mM DTT and 0.5% IPG.

### 2-DE with IPG strips

2-DE was performed as described elsewhere (Gorg et al., 2004), using precast IPG strips (pH 3–11 NL, non-linear, 24 cm length; GE Healthcare) in the first dimension (IEF). Labeled samples were combined with up to 450 μL rehydration buffer (7 M urea, 2 M thiourea, 4% CHAPS, 0.5% IPG buffer pH 3–11 NL, 40 mM DTT, and traces of bromophenol blue) and loaded onto IPG strips. Typically, 150 μg proteins were loaded on each IPG strip and IEF was carried out with the IPGPhor II (GE Healthcare). Focusing was carried out at 20°C, with a maximum of 50 μA/strip. Active rehydration was achieved by applying 50 V for 12 h. This was followed by a stepwise progression of 500 V for 500 Vh, gradient ramp from 500 to 1000 V for 1 h, gradient ramp from 1000 to 3000 V for 1 h, gradient ramp from 3000 to 5000 V for 1 h, gradient ramp from 5000 to 8000 V for 1 h, gradient ramp to 8000 V over 1 h, then 8000 V constant for a total of 44,000 Vh. After IEF separation, the strips were equilibrated twice for 15 min with 50 mM Tris-HCl, pH 8.8, 6 M Urea, 30% glycerol, 2% SDS, and a trace of bromophenol blue. The first equilibration solution contained 65 mM DTT, and 53 mM iodoacetamide was added in the second equilibration step instead of DTT. The strips were sealed on the top of the gels using a sealing solution (0.5% agarose, 0.5% SDS, 0.5 M Tris-HCl). Second-dimension SDS-PAGE was performed in Dalt II (GE Healthcare) using 1 mm-thick, 24-cm, 13% polyacrylamide gels, and electrophoresis was carried out at a constant current (45 min at 2 mA/gel, then at 15 mA/gel for ∼16 h at 20°C). Electrophoresis was completed once the bromophenol blue dye front reached the bottom of the gel. When relevant, gels were stained with ProQ Diamond (Invitrogen), a phosphorylation specific fluorescent stain or with SYPRO^®^ Ruby, and Coomassie^®^ Brilliant Blue stains for spot picking.

### Image acquisition and analysis

After electrophoresis, gels were scanned using the Typhoon Trio Imager according to the manufacturer’s protocol (GE Healthcare). Scans were acquired at 100 μm resolution. Images were subjected to automated difference in-gel analysis using Progenesis SameSpots software version 2.0.2 (Non-linear Dynamics Ltd.). The Cy3 gel images were scanned at an excitation wavelength of 540 nm with an emission wavelength of 590 nm, Cy5 gel images were scanned at an excitation wavelength of 620 with an emission wavelength of 680 nm, while the Cy2 gel images were scanned at an excitation wavelength of 532 nm with 526 nm emission filter. Gel spots were co-detected as DIGE image pairs, which were linked to the corresponding in-gel Cy2 standard. Between gels comparisons were performed utilizing the in-gel standard from each image pair using Progenesis SameSpots. All seven groups (12, 24, 36, 48, and 72 hpi of infected and normal samples were compared with each other; and fold values, as well as *p*-values of all spots, were computed with the Progenesis software, using one way ANOVA analysis. All spots were manually checked before applying the statistical criteria (ANOVA *p* < 0.05 and fold ≥1.5). The normalized spot volumes were used in statistical processing. The gels were then stored in 1% acetic acid at 4°C until spot excision.

### Protein identification

Protein spots of interest were excised from the gels, washed, in-gel reduced and S-alkylated, followed by digestion with porcine trypsin (Promega) overnight at 37°C (Shevchenko et al., 1996). The solution containing peptides released during in-gel digestion were transferred to sample analysis tube prior to mass analysis. LC/MS/MS used an integrated Agilent 1100 liquid chromatography–mass-selective detection (LC-MSD) trap (XCT-Ultra 6330) controlled with ChemStation LC 3D (Rev A.10.02). The Agilent XCT-Ultra ion trap mass spectrometer is fitted with an Agilent 1100 CapLC and nano-LC sprayer under the control of MSD trap control version 5.2 Build no. 63.8 (Bruker Daltonic GmbH). Injected samples were first trapped and desalted on the Zorbax 300SB-C18 Agilent HPLC-Chip enrichment column (40 NL volume) for 3 min with 0.1% formic acid delivered by the auxiliary pump at 4 μl/min. The peptides were then reverse eluted and loaded onto the analytical capillary column (43 mm × 75 μm ID, also packed with 5 μm Zorbax 300SB-C18 particles) connected in-line to the mass spectrometer with a flow of 600 NL/min. Peptides were eluted with a 5 to 90% acetonitrile gradient over 16 min. Data-dependent acquisition of collision induced dissociation tandem mass spectrometry (MS/MS) was utilized. Parent ion scans were run over the *m/z* range of 400–2200 at 24,300 *m/z-s*. MGF compound list files were used to query an in-house database using Biotools software version 2.2 (Bruker Daltonic) with 0.5 Da MS/MS ion mass tolerance. Protein identification was accomplished by database search using MASCOT (Matrix science, London, UK). Protein identifications were considered to be positive when the protein score was >50 (*p* < 0.05).

### Activity-based protein labeling

*S. solfataricus* proteins were labeled with previously synthesized aqueous soluble rhodamine dyes (Dratz and Grieco, 2009, 2010) coupled to (1) a phenyl vinyl sulfonate (ZG-PVS) for Tyrosine phosphatase labeling (Liu et al., 2008), (2) a fluorophosphonate (ZG-FP) for Serine hydrolase labeling (Liu et al., 1999), and (3) acyloxymethyl ketone (ZG-AOMK) with a DVED peptide sequence for caspase labeling (Kato et al., 2005). Structures are shown in Figure 5. A publication detailing synthesis and properties of the dye is forth coming. The labeling reactions were performed using each corresponding dye at a final concentration of 5 μM in PBS, pH 7.0, for 20 min at room temperature. Reactions were quenched with four-volumes of cold to precipitate the proteins. The sample precipitates were solubilized in 2D gel-loading buffer containing 40 mM DTT then loaded onto 24 cm, 3-11 NL isoelectric focusing gel strips. ABPP labeled 2D-gels were scanned on the Typhoon fluorescence imager using a standard 532/580 nm filter set. Gels were then stained with SyproRuby (Molecular Probes) to confirm equal protein loading before DIGE analysis.

## Results

Batch cultures of *S. solfataricus* (P2) grown at 80°C, pH 2.5, were infected during early log phase with STIV at a multiplicity of infection (MOI) of ∼10. Infected and control cultures were treated identically and subsamples were removed at 12, 24, 36, 48, 60, and 72 h post inoculation (hpi). Cell growth rate was only moderately slower in the inoculated cultures as monitored by optical density (Figure S1 in Supplementary Material). Epifluorescent viral counts (Figure S1 in Supplementary Material), qPCR for the viral genome, and an ELISA specific for the major capsid protein of STIV (data not shown) were used to confirm viral replication (Ortmann et al., 2008). Together this data suggested that a single round of viral replication occurred. Cell counts and electron microscopy indicated that approximately 10% of the SsP2 cells became infected (Ortmann et al., 2008).

Cells from three biological replicates of infected and control cultures were subjected to standard minimal CyDye labeling and then analyzed by 2D-DIGE (Tannu and Hemby, 2006). Greater than 1000 spots were found on each gel and after filtering to remove irregularities, 700 were used in the analysis across all gels (Figure S2 in Supplementary Material). Using an approximate twofold change cutoff, there were 71 regulated proteins with 40 being more abundant and 31 less abundant in the infected samples. Changes in protein abundance began at 12 hpi and peaked near 48 hpi. The general trend was an immediate down regulation of host proteins followed by increasing up regulation over the course of the infection. Proteins spots were identified using in-gel digestion and LCMS/MS analysis, followed by searching of a local MASCOT database containing all viral and host ORFs greater than 50 amino acids. This expands the database slightly beyond what has been annotated in TIGR and NCBI. Previous experience with STIV indicated that non-annotated reading frames can be used (Maaty et al., 2006), therefore, this approach was adopted to minimize false negatives in the preliminary IDs. A list of the time points, identified host proteins, and associated COG groups is shown in Table 1. More detailed information including spot location, fold change, sequences, scores, percent coverage, and links to genome databases and annotation is provided in Table S1 in Supplementary Material.

**Table 1:** **2D-DIGE identified proteins organized within COGs**.

COG functional category	Protein and regulation
Unclassified	CRISPR-associated regulatory protein, Csa2 family (csa2), SSO1442 (48)↑; CRISPR-associated protein, Csa5, SSO1443 (36,48)↓; CRISPR-associated protein, TM1791.1, SSO1988 (36,48)↓
^§^Energy production and conversion	Acetyl-CoA synthetase, SSO1111 (36,72)↑; Heterodisulfite reductase subunit B, SSO1129 (72)↑; Heterodisulfide reductase subunit C, SSO1134 (48, 72)↑; FkbR2, putative, SSO1135 (48,72)↓; Carbon monoxide dehydrogenase small chain, SSO2433 (12,24,36,48,72)↓; Succinyl-CoA synthetase alpha subunit, SSO2482 (36)↑; Succinyl-CoA synthetase beta subunit, SSO2483 (12, 24, 48, 72)↓; Rubrerythrin, SSO2642 (12,24,36)↓; Electron transfer flavoprotein, SSO2817 (48)↑
Amino acid transport and metabolism	X-pro aminopeptidase, SSO0010 (12,24)↓; Prolidase, SSO0363 (48,72)↓; d-3-phosphoglycerate dehydrogenase, SO0905 (36)↑; FkbR2, putative, SSO1154 (12,24,36,48)↓; *O*-succinylhomoserine (thiol)-lyase, SSO2368 (72)↑; 3-isopropylmalate dehydratase, SSO2471 (36)↑
Nucleotide transport and metabolism	Uracil phosphoribosyltransferase, SSO0231 (48)↑, Glutamine amidotransferase, SSO0571 (72)↑
Carbohydrate transport and metabolism	Phosphomannomutase, SSO0207 (12, 24)↓(48)↑, phosphoenolpyruvate synthase, SSO0883 (12,24)↓; bifunctional phosphoglucose/phosphomannose isomerase, SSO2281(72)↑; ABC transporter, SSO2850 (72)↑; Fructokinase, SSO3195 (36, 48)↑
Lipid transport and metabolism	Acetyl-CoA acetyltransferase, SSO2061 (12, 24)↓(48)↑, Lipase, SSO2493 (36,48)↑, Acyl-CoA dehydrogenase, SSO2511 (72)↑
^§^Translation	50S ribosomal protein L7Ae,SSO0091(48)↑; Methionine aminopeptidase, SSO0098 (36,48)↑; Aspartyl-tRNA synthetase, SSO0173 (72)↑; LSU ribosomal protein L12AB, SSO0342 (48)↓; LSU ribosomal protein L11AB, SSO0346 (48)↓; Threonyl-tRNA synthetase homolog, SSO0384 (72)↑; SSU ribosomal, SSO0411 (48)↑; prolyl (glutamyl) tRNA synthetase, SSO0569 (72)↑; ribosomal protein S5, SSO0698 (48)↑
^§^Transcription	30S ribosomal protein S14 homolog, SSO0049 (12)↓; transcription elongation factor NusA-like protein, SSO0172 (72)↓; Transcription factor E, SSO0266 (12,24,72)↓; DNA binding protein SSO10b, SSO0962 (36)↓; Transcriptional regulator marR family, SSO1082 (12)↑; transcriptional regulator Lrs14, SSO1108 (36)↓; Regulatory protein; AsnC family, SSO5522 (12)↑
Replication, recombination, and repair	DNA repair protein radA, SSO0250 (36,48)↑; Endonuclease IV-like protein, SSO2156 (48)↑, Single-stranded DNA binding protein, SSO2364 (48,72)↓
^§^Post-translational modification, protein turnover, chaperones	Thermosome beta subunit, SSO0282 (12,24)↓(48)↑; prefoldin subunit alpha, SSO0349 (72)↓; Prefoldin beta subunit, SSO0730 (12,24)?; proteasome alpha subunit, SSO0738 (36,48)↑; thermosome subunit alpha, SSO0862 (36,72)↑; Peroxiredoxin, SSO2613 (36,48,72)↓; Thermosome gamma subunit, SSO3000 (12,72)↓
Inorganic ion transport and metabolism	Superoxide dismutase, SSO0316 (12,24)↓(36,48)↑
General function prediction only	Molybdenum transport protein ModA related protein, SSO1066 (36)↑; Carbon monoxide dehydrogenase, large chain, SSO1209 (24)↓; 3-oxoacyl-(acyl carrier protein) reductase (fabG-4), SSO2205 (72)↑; 3-oxoacyl-(acyl carrier protein) reductase (fabG-5), SSO2276 (48)↑; MoxR-like ATPases, SSO2363 (12,24)?; Carbon monoxide dehydrogenase, medium chain, SSO2434 (36, 48)↑; NAD-dependent alcohol dehydrogenase, SSO2536 (72)↑, INOSINE-5-MONOPHOSPHATE DEHYDROGENASE putative, SSO2588 (12,24)↓; Carbon monoxide dehydrogenase, medium chain, SSO2636 (36)↑; Alcohol dehydrogenase (adh-13), SSO2878 (36)↑; Tryptophan repressor binding protein, SSO3155 (72)↑
Function unknown	Hypothetical protein, SSO0276 (12)↑, Hypothetical protein, SSO0286 (36)↑; Conserved hypothetical protein, SSO1098 (12,24,36,48)↑; Hypothetical protein, SSO2569 (48)?; Conserved hypothetical protein, SSO2749 (12,24)↓
Signal transduction mechanisms	Universal stress protein, SSO1865 (12,24,36,48)↓

*Numbers in parenthesis designate time points (hpi). ^§^Majority of regulated proteins belongs to those COG groups. ↑↓ Arrows indicates direction of regulation*.

*The following COG functional categories are not detected in this study. RNA processing and modification, coenzyme transport and metabolism, chromatin structure, and dynamics, cell cycle control, mitosis, and meiosis, cell wall/membrane biogenesis, cell motility, secondary metabolites biosynthesis, transport, and catabolism, intracellular trafficking and secretion, defense mechanisms, extracellular structures, nuclear structure and cytoskeleton*.

**Figure 1:**
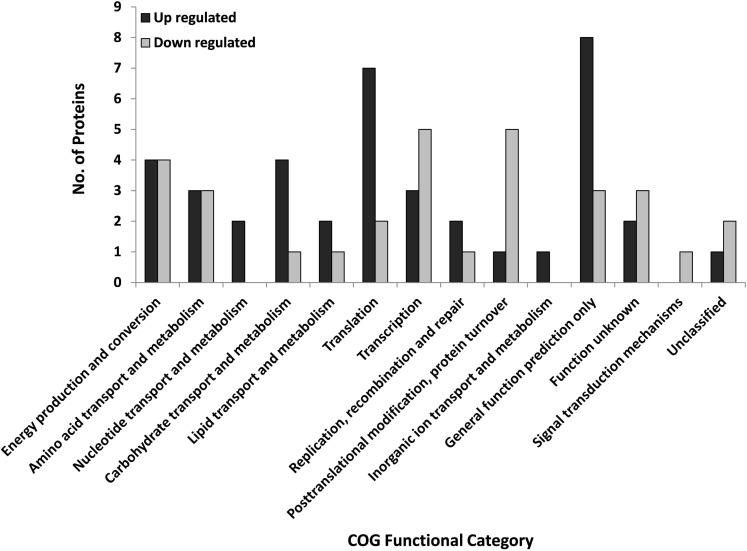
**Regulated *S. solfataricus* (P2) proteins by COG designation that were regulated during STIV infection**. Proteins were classified as regulated if the fold change at any time point was ∼twofold and *p* < 0.05 (ANOVA). There were 40 up-regulated and 31 down regulated proteins.

Based on the COG partitioning, the regulated proteins fell into 14 of the 26 groups (Figure 1; Table 1). More than one-half of the regulated proteins were in just five of the COG groups (Transcription K; Translation J; PTM, protein turnover, chaperones O; Energy production and conversion C; Amino acid transport, and metabolism E). These five COG groups account for only 25.4% of the *S. solfataricus* (P2) genome; therefore they are over-represented in the regulated protein fraction. Eight of the 71 proteins (SSO: 0286, 730, 1098, 1442, 2749, 1443, 1988, and 2569) fell into the unknown function (COG = S) and unclassified categories. At the genome level 49% of *S. solfataricus* (P2) genes are currently assigned to a specific category while 27% of the open reading frames have no assigned functional role and are listed as hypothetical genes. The remaining 24% are listed as conserved hypothetical genes. Therefore, the proteins regulated during viral infection tend to be in familiar COG groups or have sequence homology with known proteins, rather than being novel proteins unique to *Sulfolobus* or Archaea.

A number of protein functional categories displayed a mixed response to infection with some members increasing in abundance and others decreasing (Figure 1; Table 1). For example, proteins with a role in transcription and translation were split between up and down categories. Of particular interest is ribosomal protein L7Ae. It plays a critical role in binding and folding of small RNAs (Dennis and Omer, 2005; Omer et al., 2006), which are playing an ever expanding biological role. Another functional group of proteins with both up and down regulation were those involved with carbohydrate metabolism and transport, two processes important to STIV replication (Larson et al., 2006; Maaty et al., 2012).

Only two STIV proteins, B345 (major capsid protein) and C92 (formation of VAPs) were detected by the DIGE analysis, both at 36 hpi. B345 and C92 are expressed in large quantities during viral replication (Maaty et al., 2012), so it is reasonable that when viral replication is occurring in 10% of the population, only the most abundant virus proteins would be detected. B345 was identified in more than one horizontally displaced spot on the gels, indicative of several different PTM isoforms. Similar multiple isoforms were found in purified STIV particles (Maaty et al., 2006) and from studies using the readily infectable isolate, SsP2-2-12 (Maaty et al., 2012). The other viral protein, C92 (9.8 kDa, pI 5.19) is a small membrane protein that assembles into the large VAPs that have been described recently (Brumfield et al., 2009; Snyder et al., 2011). The mechanism behind this polymerization is currently unknown and no similar structures have ever been reported beyond STIV and SIRV2.

As the number of genomic and proteomic studies characterizing cellular response to various perturbations have accumulated, features of a general response have emerged. Common participants in the stress response include heat shock proteins, chaperones, and mediators of oxidation/reduction (Macario et al., 1999; Kvint et al., 2003; Casado-Vela et al., 2006; Chertova et al., 2006). A number of the regulated proteins found in this study are consistent with a general stress response following infection. Superoxide dismutase (SSO0316) participates in the scavenging of reactive oxygen species and has been shown to be up-regulated in animal and plant cells during viral infection (Alfonso et al., 2004; Casado-Vela et al., 2006). In contrast, down regulated proteins include SSO1865, which is homologous to universal stress protein (USP), and Rubrerythrin (SSO2642), a non-heme iron binding protein found in many air-sensitive Bacteria and Archaea that is a member of a broad superfamily of ferritin-like diiron-carboxylate proteins (Andrews, 1998; Kurtz, 2006).

### Antiviral systems

Inoculation of *S. solfataricus* P2 cultures with high MOI of STIV leads to viral production and eventual cell lysis only in a limited number of cells, ∼10% (Ortmann et al., 2008). The reason for this is unknown, although such fractional susceptibility to infections has also been confirmed with other archaeal virus/host systems (Prangishvili et al., 1999; Kessler et al., 2004; Peng et al., 2004). Plausible explanations include, but are not limited to (i) not all of the cells express the viral receptor, (ii) cellular host factors required for replication are at low levels or possibly are missing, or (iii) an antiviral defense system is functioning in most but not all cells. Members of the CAS family were among the regulated host proteins. These CAS family are part of the CRISPR/Cas (clusters of regularly interspaced short palindromic repeat) prokaryotic adaptive immune system which help prokaryotes resist viral infection (Barrangou et al., 2007). CRISPR regions have been identified in *Sulfolobus* spp. DNA (Mojica et al., 2005; Kunin et al., 2007) and specific matches to viral sequences, including STIV, are present (Mojica et al., 2005). Three CRISPR-associated proteins (CAS proteins) SSO 1442, 1443, 1988 were the significantly regulated group of proteins. The presence of six additional CAS proteins was confirmed by survey-based spot picking (SSO: 1398, 1399, 1401, 1441, 1514, and 1991) bringing the total to nine. Recent studies of the CRISPR/Cas system in *S. solfataricus* (Garrett et al., 2011a,b), including structural studies of the (CRISPR)-associated complex for antiviral defense (CASCADE) that targets invading DNA (Lintner et al., 2011b), the CMR complex that targets invading RNA (Zhang et al., 2012), and potential transcriptional regulators of CRISPR/Cas (Lintner et al., 2011b) are beginning to shed light on how this system works in Archaea. It is interesting to note that prior study using a fully STIV susceptible strain of *S. solfataricus* did not detect expression of any CAS proteins (Maaty et al., 2012).

### Post-translational modification of host proteins

**Figure 2:**
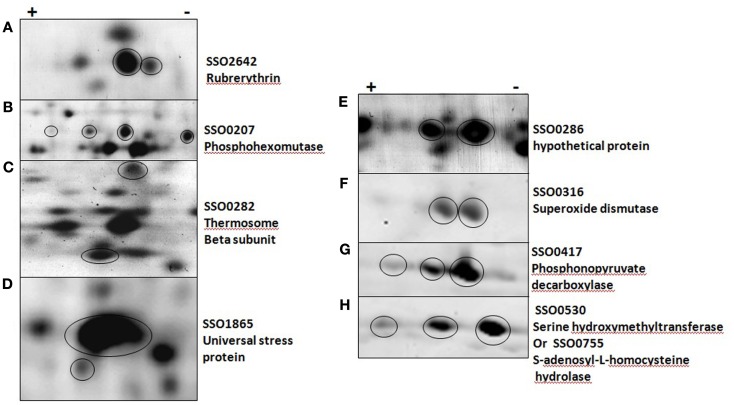
**Post-translationally modified proteins in five *solfataricus* (P2)**. **(A–D)** were imaged with a general protein stain, **(E–H)** with a phosphoprotein specific stain. Circles indicate protein of interest in each panel.

Protein synthesis and degradation are not the only ways in which protein activity can be regulated. PTMs are a common and often rapid way in which protein activity can be altered. Based on gene annotation and functional characterization, the presence of enzymes involved with the addition and removal of PTMs such as methylation, acetylation, phosphorylation, glycosylation, and N-terminal processing are present in *S. solfataricus* (P2; Krishna and Wold, 1993; She et al., 2001; Chaban et al., 2006). Finding the targets and frequency of PTM is a challenging task and currently, little is known about the use of PTM in Archaea (Barry et al., 2006). The ability to detect PTMs and changes in PTMs globally, at the level of the proteome, without limiting the scope of the experiment, is a major strength of the 2D-DIGE approach. Changes in protein pI and/or MW are caused by most PTMs leading to altered isoform positions on the gel and changes in spot intensities. Proteins with less than complete modification at a specific site will be found in more than one spot and the spot intensity ratios can be used to follow the fraction of modification and the kinetics of modification. Of the 71 regulated proteins, 23 were identified in more than one spot, suggesting that regulation may be the result of changes in PTMs and corresponding shifts on the gels (see Figure 2). A number of proteins appear in 2–6 horizontally shifted spots, indicating the presence of multiple modifications.

Protein phosphorylation is a common PTM that changes the pI of the intact protein and shifts the protein position on the 2D gel. The detailed characterization of specific protein phosphorylation has only been described for a few *Sulfolobus* proteins. Interestingly, one of these, phosphohexomutase (SSO0207; Ray et al., 2005) was among the regulated proteins. Phosphohexomutase was found in six different gel spots and exhibited a complex pattern of regulation (Figure 2B). Rubrerythrin, the putative stress response factor discussed above, was found in multiple horizontal spots that were down regulated at 12 and 24 hpi in addition to vertical shifts. Shifts in the vertical migration (molecular weight dimension) can be minor as seen with USP (Figure 2D), or major as in the case of the thermosome beta subunit (Figure 2C). Another protein known to be regulated by phosphorylation is d-gluconate dehydratase (SSO3198), a key enzyme in the non-phosphorylated Entner–Doudoroff pathway (Kim and Lee, 2005). We found this to be up-regulated at 48 h and to appear in multiple positions on the gels. All of these PTM were detected using standard CyDye DIGE analysis, illustrating a strength of the 2D-DIGE method.

**Figure 3:**
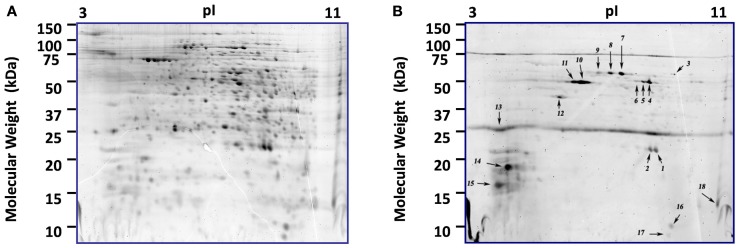
**Comparison of phosphorylated and total proteome of *S. solfataricus* (P2)**. 2D-gels at 36 h post-infection. **(A)** Proteome staining with the fluorescent dye Sypro Ruby. **(B)** Same gel using the phosphoprotein specific stain, ProQ diamond. Each of the numbered spots was picked and the proteins were identified by LCMS and results are shown in Table S4 in Supplementary Material.

Directed PTM experiments using ProQ Diamond, a phosphorylation specific fluorescent stain were conducted to look specifically for phosphorylated proteins. 2D-gels of control and infected cells at 36 and 48 hpi were analyzed after ProQ Diamond staining and ∼100 protein spots could be detected, 18 of which were intense (Figure 3). The reported detection limit for this dye is 1–16 ng/band (Invitrogen). Fifteen of the 18 intense spots contained a single protein (Table S3 in Supplementary Material). The other three spots contained more than one protein, each with good sequence coverage, so the specific protein responsible for ProQ Diamond staining of the spot could not be determined. Two of the 18 intense ProQ Diamond staining spots appeared only after infection; ribosomal protein L22AB (SSO0713) and geranylgeranyl hydrogenase (SSO2353). The latter protein was also found in one of the only other proteomic surveys of *S. solfataricus* (P2), however the presence of phosphorylation was not addressed (Barry et al., 2006). The only other documented report of phosphorylation for one of the regulated proteins concerned the conserved hypothetical protein (SSO0286), which was found here in two spots and has 93% sequence similarity to Fructose-1,6-Bisphosphatase from *S. tokodaii* (Nishimasu et al., 2004). Phosphorylation of Fructose-1,6-Bisphosphatase is typically involved in switching between glycolysis and gluconeogenesis, which can be of value when cellular energy is needed but the glucose level in the medium is low. Five of the proteins with significant phosphorylation were also in the group of 71 regulated proteins discussed above.

### Network regulation

**Figure 4:**
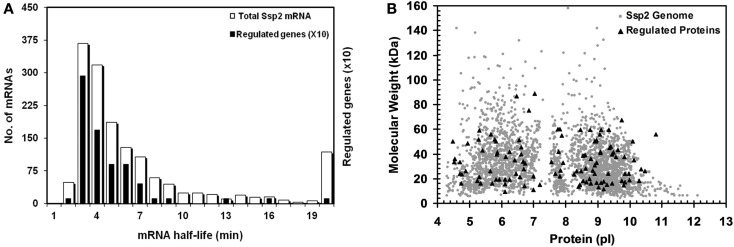
**mRNA half-life and MW/pl distribution of proteins with altered abundance**. **(A)** Half-life of 1880 *S. solfataricus* (P2) mRNAs (white bars) and those for regulated proteins (black bars). Regulated proteins have RNA half-lives that are significantly biased against slow turnover based on a Kolmogorov–Smirnov test (*p*=0.0002). Bars for the regulated proteins are shown 10X for clarity; **(B)** Virtual 2D gel of *S. solfataricus* (P2) proteome. Calculated MW and pi distribution for the regulated proteins (triangles) and all predicted *S. solfataricus* (P2) genes (grey circles). The regulated proteins were found to be biased with respect to pi (*p*=0.001) but not MW (*p*=0.41) based on a Kolmogorov–Smirnov test.

The analysis of the STIV-*S. solfataricus* (P2) interaction has focused on specific proteins and pathways above. A more general analysis of the data may also be useful for putting the data in perspective with other organisms and approaches. Data on mRNA half-life in *S. solfataricus* and *S. acidocaldarius* were previously measured in a genome-wide study (Andersson et al., 2006). The results were essentially the same for the two species, with a median half-life of ∼5.3 min and ∼50% of the mRNA lifetimes between 4–8 min. We compared the half-life of mRNAs corresponding to proteins that were differentially regulated following virus infection with the genome-wide values (Figure 4A). The observed pattern shows that regulated proteins come from mRNAs with a shorter than average half-life. Analysis of the lifetime data based on the 62 regulated proteins for which mRNA half-lives were known and 1961 half-life values from *S. solfataricus* using the Kolmogorov–Smirnov test, confirmed that the distributions were different (*p* = 0.0002). The study by Andersson et al. found that functional categories for translation, metabolism, and energy production were over-represented in the shortest half-life group of mRNAs. This is consistent with the population of proteins that were found to be regulated in the present study. The bias toward proteins that come from mRNAs with rapid turnover being regulated proteins is also consistent with computational models using a cost-benefit analysis for interpreting gene network regulation (Carlson, 2007).

An important consideration with any “omics” approach is data bias. It is commonly assumed that 2D gel analysis is biased against proteins at the pI and MW extremes. To test this, we compared the isoelectric point and apparent mass of the regulated *S. solfataricus* (P2) proteins to those of the predicted proteome. For visualization, the pI and MW of all annotated *S. solfataricus* (P2) genes were plotted as a virtual 2D gel, overlayed with darker spots for the regulated proteins (Figure 4B). Analysis of the 71 regulated proteins and the ∼3000 predicted genes, using the Kolmogorov–Smirnov test, revealed that there was no bias of the regulated proteins with respect molecular weight (*p* = 0.41). However, the regulated proteins did show a bias with respect to pI (*p* = 0.001). At this point, it is not known whether this is due to technical issues related to the 2D gel process or is a reflection of the chemical properties of regulated proteins. However, this result is interesting because, while there is no inherent cost-benefit basis for selectively altering the turnover of mRNA’s that encode for proteins with different pIs, there clearly is for extremes in MW. This is because the synthesis of short and long mRNAs and proteins require different resource commitments that will be allocated differently during stress response (Andersson et al., 2006; Carlson, 2007).

### Activity-based protein profiling

**Figure 5:**
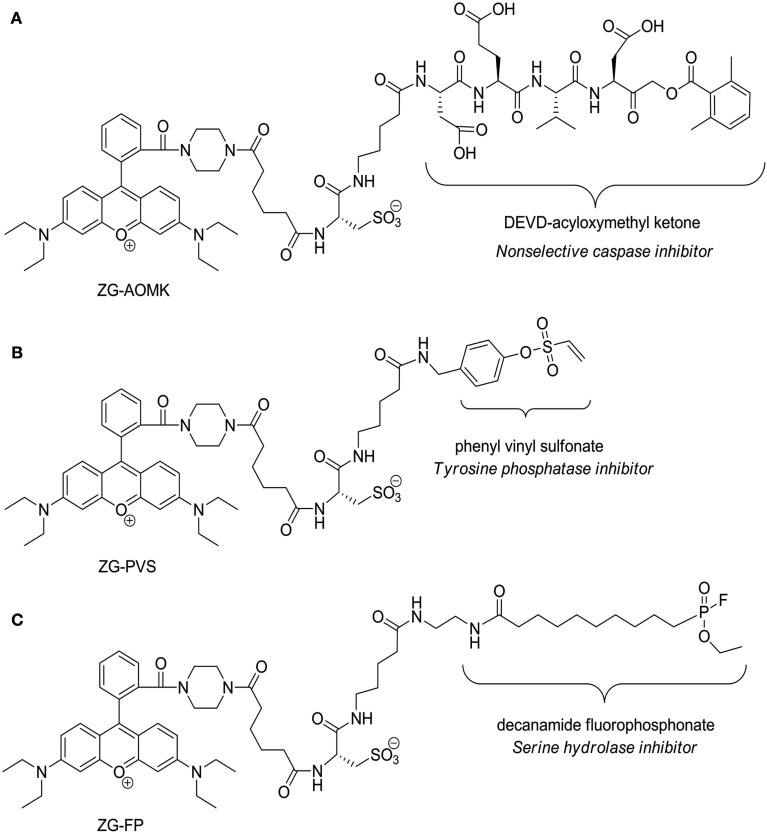
**Structures of the zwitterionic ABPP probes**. **(A)** Acyloxymethyl ketone probe (ZG-AOMK) for caspase. **(B)** Phenyl vinyl sulfonate probe (ZG-PVS) for Tyrosine phosphatase. **(C)** Fluorophosphonate (ZG-FP) for Serine hydrolase. Note that the protein reactive functional groups are labeled and the protein classes to which they inhibit are denoted below.

A commonality of genomic and proteomic methods is that each measures the amount of a biomolecule present in a cell at a given time. While this can be highly informative and is the basis for nearly all studies looking at mRNA and proteins, the amount of mRNA and protein are proxies for biological activity. In contrast, ABPP is a powerful analytical method to detect and compare protein activities across proteomes. The use of ABPP in general is increasing rapidly, but few probes have been synthesized that demonstrate compatibility with 2D-Gel analysis (Schicher et al., 2010). The majority of standard fluorescent dyes behave poorly on 2D-gels because they alter protein pI, decrease solubility, and/or result in smearing. We have synthesized a series of dyes specifically designed for optimal 2D gel analysis, that exhibit high aqueous solubility and do not perturb protein pI (Dratz and Grieco, 2009, 2010), three of which are presented here as activity-based probes for the first time (Figure 5). This is also the first survey of caspases, serine hydrolases, and tyrosine phosphatases in an archaeal organism.

#### Caspase-like enzymes

**Figure 6:**
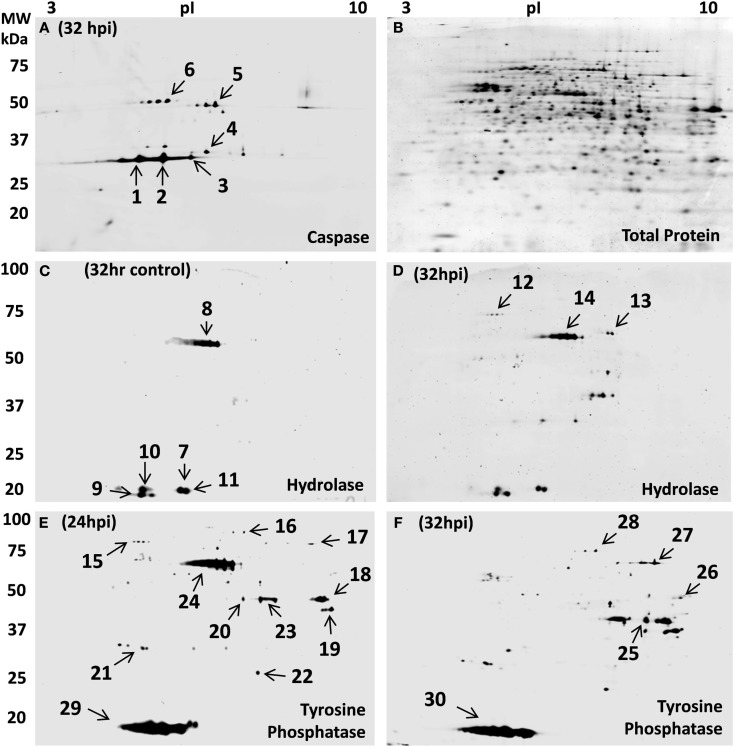
**Host protein activity-based reactivity changes during STIV infection**. **(A)** Novel zwitterionic acyloxymethyl ketone probe (ZG-AOMK) for caspase as described in the text, was reacted with total soluble protein from 32 h infected *S. solfataricus*. Approximately 27 protein spots were labeled with the caspase specific probe. **(B)** Subsequent staining for total protein, using SyproRuby, showed ∼800 spots. Zwitterionic fluorophosphonate (ZG-FP) for Serine hydrolase was reacted with total soluble protein from *S. solfataricus* control **(C)** and 32 hpi **(D)**. Approximately 15 protein spots were labeled with the Serine hydrolase specific probe. Phenyl vinyl sulfonate probe (ZG-PVS) for Tyrosine phosphatase was reacted with total soluble protein from virus infected *S. solfataricus* 24 hpi **(E)** and 32 hpi **(F)**. Subsequent staining of gels in **(C–F)** for total protein, using SyproRuby, showed ∼800 spots (as shown in **B**).

Caspases are a family of cysteine proteases that regulate programmed cell death (apoptosis) and other functions in eukaryotic cells. Caspases are present in cells as zymogens that require activation cascades (Boatright and Salvesen, 2003). The presence of caspase-like proteins in Archaea was unknown until a report of caspase eight antibody cross-reactivity in *Haloferax volcanii* (Bidle et al., 2010). Genomic analysis of *H. volcanii* identified a subset of 18 potential target proteins containing the signature tetrapeptide caspase cleavage motif (IETD), however caspase-like function was not assessed in the study. Therefore, we used a fluorescently tagged caspase specific probe (ZG-AOMK) to screen for caspase-like enzyme activity. Incubation of total soluble proteins from infected *S. solfataricus* (P2) cells with ZG-AOMK followed by 2D gel analysis, revealed groups of spots between 30–75 kDa with significant labeling (Figure 6A).

Protein staining of the gel (Figure 6B) showed that the labeling was highly discrete, because the activity and protein abundance patterns were quite distinct. Six spots were selected for in-gel proteolysis and LCMS identification, four of which contained proteins known to have reactive cysteines. Thiosulfate sulfurtransferase (cysA-2), SSO1817, was in spot 4. This enzyme contains a Rhodanese Homology Domain with a catalytically active cysteine residue. Spot 6 had 5-Oxo-l-prolinase, which has a sulfhydryl involved with NTPase and prolinase activities (Williamson and Meister, 1982). Adenosylhomocysteinase, spot 5, has a cysteine in the catalytic center that is reported to maintain reduction potential for effective release of the reaction products and regeneration of the active form (NAD+ form) of the enzyme (Yuan et al., 1996). Lastly, 4-aminobutyrate aminotransferase, spot 1, has a pair of cysteines that mediate dimerization (Kim and Churchich, 1989). Caspase inhibitors are well known to be promiscuous, reacting with enzymes having cysteines in the active site (Furman et al., 2009). The labeling of 4-aminobutyrate aminotransferase was a bit more puzzling, however, on closer inspection, it was noted that a cysteine is involved with dimerization. Specificity of the protein–protein interaction must then be an inherent part of the region surrounding the active cysteine. Making it much like a caspase in that it recognizes a specific amino acid sequence and places a reactive cysteine in position to react. A detailed list of the proteins labeled with the caspase probe can be found in Table S4 in Supplementary Material.

#### Serine hydrolases

The second activity probe used targeted serine hydrolases which are one of the largest and most widely distributed enzyme classes in all three kingdoms of life. This class of enzymes utilizes a conserved serine nucleophile to hydrolyze amide, ester, and thioester bonds in both protein and small molecule (metabolite) substrates. A broad spectrum fluorophosphonate probe has been shown to effectively modify the serine hydrolase superfamily including proteases, lipases, esterase, acetylcholinesterase, thioesterases, some phospholipases, and amidases (Liu et al., 1999; Simon and Cravatt, 2010). Biochemical, structural, and *in silico* studies have confirmed the presence of the serine hydrolase superfamily members mentioned above as well as α-amylases (Janecek and Blesak, 2011) and prolyl oligopeptidases (Bartlam et al., 2004) in Archaea. We used the fluorophosphonate (ZG-FP) probe (Figure 5) to test for such activity, and labeled total soluble protein during viral infection. *S. sulfolobus* (P2) samples at 32 control and 32 hpi were incubated with ZG-FP and then analyzed by 2D-gels with differential labeling of total protein. Approximately ∼15 spots at both control and 32 hpi were specifically tagged (Figures 6C,D), the pattern was significantly different between the two gels. The protein spots were identified by in-gel digestion and LC/MS/MS. Fifteen different proteins were identified including gene annotated as metal-dependent hydrolase, serine hydroxymethyltransferase; *S*-adenosylhomocysteine hydrolase (see Table S4 in Supplementary Material for complete list). This approach promises to be a widely applicable tool for better understanding pathways that are regulated during infection and for many other functional protein annotation projects.

#### Tyrosine phosphatases

The third probe addresses protein dephosphorylation. The level of protein phosphorylation is balanced by the antagonistic functions of protein kinases and phosphatases (Denu et al., 1996). Tyrosine phosphatases remove phosphate groups from the amino acid tyrosine and are very important component in eukaryotic and bacterial signaling pathways (Li and Dixon, 2000; Tonks and Neel, 2001). *In silico* analysis of archaeal organisms found several candidate phosphatases (Stravopodis and Kyrpides, 1999), and the18 kDa tyrosine/serine phosphatase SSO2453 which was previously described in *Thermoeoeeus kodakaraensis* (Jeon et al., 2002). Another group of low molecular weight protein-tyrosine phosphatase are VH1-like phosphatases which have been found in Bacteria, Archaea, plants, yeast, insects, worms, and mammals. A homolog to this phosphatase is annotated in *S. solfataricus P2* as tyrosine phosphatase (SSO2453). Their average size of 16 kDa, is close to the major low molecular weight spots 29 and 30 in Figures 6E,F. The presence of phosphorylated proteins (Figure 3) suggested that phosphatases may play a role in signaling during STIV infection. To conduct a biochemical test, control and STIV infected cells were compared using 2D-gels with differential labeling of protein and enzyme activity 24 and 32 hpi after reaction with ZG-PVS. As with the other two probes, selective labeling of proteins was evident and differences were observed between the samples. Two regions showed very strong labeling (spots 24 and 29, Figure 6E). Protein identification of spot 29 from three gels consistently revealed rubrerythrin (SSO2642) and prefoldin (SSO0730). In addition, an 11.5 kDa acylphosphatase, SSO0887, which closely matches the position on the gel was identified. The signal from the probe was very strong (Figure 6E), even though the amount of protein was low, indicating that the reactivity was high. In-gel proteolysis and mass spectrometry analysis only found a single peptide, but it occurs at the pI and MW expected, the identification score for the peptide was highly significant, and the protein is small, limiting possible peptides to be detected. Therefore, we propose the SSO0887 is the actual protein being labeled.

The other region with intense labeling (spot 24) contained three proteins (2-methylcitrate dehydratase, serine hydroxymethyltransferase, and *S*-adenosyl-homocysteine hydrolase) none of which would be expected to react. There could be a mis-annotation, or we failed to detect the protein responsible for the labeling. None of the other spots provided hits to proteins with direct linkage to phosphatases (the complete list of identified proteins can be found in Table S4 in Supplementary Material). This inspired a second sequence-based check for potential phosphatases in *S. solfataricus* P2. Blast alignments of phosphatase sequences from other archaeal organisms lead to the putative identification of four additional phosphatases (NagD-like sugar phosphatase SSO2355, phosphoglycolate phosphatase SSO0094, phosphohexomutase SSO0481, and a tyrosine phosphatase SSO2453). The question now is what biological role is being carried out by the acylphosphatase in spot 29 and also, which protein is being labeled in spot 24 that is highly reactive at 24 hpi, yet disappears entirely 8 h later. What is clear is how this probe quickly revealed that proteins with phosphatase-like activity were present and that the use of such probes can help to focus bioinformatics searches.

## Discussion

Despite widespread interest in archaeal organisms and more recently the viruses that infect them, many basic questions remain to be answered regarding their biology and biochemistry. Here we have coupled the well tested approach of following a host during viral infection/replication with multi-tiered “omics” experiments to learn more about the virus and it’s interaction with the host. Combining data on mRNA lifetime and protein abundance, protein PTMs, and protein activities, provided insights into host and virus biology. Specifically, the interplay between STIV and *S. solfataricus* (P2) was characterized during a single round of replication. The majority of the cells in this ATCC stock were resistant to infection. This was confirmed visually and with growth curves (Figure S1 in Supplementary Material). Similar findings have been reported for other archaeal viruses and specific host species (Kessler et al., 2004; Peng et al., 2004; Happonen et al., 2010). Currently, it is unclear why many of the cells in these populations appear to be refractory to viral infection and this is the first report to address infection in a population of archaeal cells in which the majority does not show signs of viral burden. Unexpectedly, a stronger host response to viral infection at the proteome level was measured when infection rate was low, compared to the clonal isolate SsP2-2-12 that is highly susceptible to infection (Maaty et al., 2012).

Viral production is much greater from a culture of the highly susceptible *S. solfataricus* (SsP2-2-12) compared to the stock *S. solfataricus* (P2) ATCC strain used in this study. Comparative proteomics confirms this, as viral proteins were readily detected on 2D-gels using DIGE analysis at 24 and 32 h post exposure (Maaty et al., 2012), in contrast to the data presented here where only the two most highly expressed viral proteins, B345 (major capsid protein) and C92 (VAP), were detected on the gels. In stark contrast to viral protein production, only ten host proteins changed in abundance in the susceptible isolate, whereas 71 host proteins from 33 different pathways (Table S2 in Supplementary Material) changed in abundance across the time course shown here for *S. solfataricus* (P2; Table S1 in Supplementary Material). This discrepancy cannot be attributed to variation in the biological replicates, or dramatically different numbers of virus per cell (Ortmann et al., 2008). Together, these results indicate that the proteomic response observed here is predominantly coming from cells that are not showing visual or growth related signs of infection suggesting that an active response to STIV may be occurring.

### Host regulated proteins

In the regulated protein pool, known stress response proteins were found, but the most intriguing host proteins; SSOs 1442, 1443, and 1988; are those from the CAS family. Proteins SSO1442 and perhaps 1443 (csa2 and csa5 respectively) are part of the CASCADE complex in *S. solfataricus* (P2) and thus play an important role in the newly characterized antiviral response in Archaea (Lintner et al., 2011b). This antiviral system uses CRISPRs as part of a double-stranded DNA monitoring system in prokaryotes (Barrangou et al., 2007). In addition to the regulated CAS proteins, six others were detected, with SSO1399, 1401, and 1441 being part of the same CASCADE complex (Lintner et al., 2011b). It has been suggested that Csa3 (Sso1445), may act as a transcription factor to regulate expression of these other CRISPR/Cas components, and that this may occur in response to viral infection (Lintner et al., 2011a), which is consistent with our results. Significantly, no regulated or constitutively expressed CAS proteins were found in the study using the readily infected SsP2-2-12 isolate (Maaty et al., 2012). This is a particularly exciting finding in that we now have a viral system and specific protein targets to test the mechanism of CASCADE *in vivo*.

### Post translation modifications

Our initial proteomics approach focused on changes in protein abundance, leading to the identification of a number of proteins and pathways found to be important in prokaryotic and eukaryotic response to viral infection (Table S1 in Supplementary Material). A distinct advantage of the 2D-DIGE approach over strictly mass spectrometry-based methods (on protein digests), is the ease with which PTMs and changes in PTMs can be detected and accurately quantified on intact proteins. Complex changes in the level of even multiple PTMs can be tracked on intact proteins and measured globally in a straight-forward manner using protein isoform abundances (Figure 2) or specific tagging for a PTM (Figure 3). It is worth stressing the point that without prior knowledge and focused methods, obtaining data on the relative populations of given protein isoforms, such as shown for phosphohexomutase and phosphonopyruvate decarboxylase (Figures 2B,G) would be extremely challenging. A clear direction for future investigation is to characterize specific targeted PTMs that are associated with biological mechanisms of interest and their functional outcomes.

### Protein to mRNA correlation

A large number of studies, including our own work on *S. solfataricus*, have detailed the lack of correlation between changes in mRNA and protein levels (Lee et al., 2003; Mehra et al., 2003; Neumann et al., 2004; Maaty et al., 2009, 2012). This is due in part to differences in the turnover rates (half-life) of a given mRNA and the protein it codes for (Taniguchi et al., 2010). Genes encoding proteins that require tight regulation, such as signaling or metabolic pathways, generally have mRNAs with shorter than average half-life (Andersson et al., 2006). Using the work of Anderson et al. as a reference, we show that the regulated proteins come from mRNAs with a shorter than average half-life. This observation could be considered within the context of a cost-benefit analysis of biomolecule synthesis (Carlson, 2007) and systems biology modeling of regulatory networks and theory (Singh and Hespanha, 2009; Babu, 2010). Broadly, this indicates that regulation in *S. solfataricus* is controlled at multiple levels and that the overall network structure is consistent with that in Bacteria such as *E. coli*.

A significant challenge in studies using transcriptomics and proteomics approaches is connecting the data to biological mechanisms. For example, as discussed above, mRNA and protein abundance have only a weak correlation in most cases. An additional separation is that protein abundance is only a very imperfect proxy for activity, as other levels of regulation exist as well. This is what makes the chemical biology approach of ABPP so exciting. For the classes of enzymes that can be targeted with this approach, the read-out is a direct measure of biological activity. When ABPP is conducted with proteomics, enzyme class screening becomes a way to broadly screen activity yet retain specificity down to the level of isozymes. Until now, the ABPP proteomics connection has relied almost entirely on 1D SDS-PAGE or affinity purification using biotinylated probes. We have adapted 2D gel-friendly dyes (Dratz and Grieco, 2009, 2010) to 2D ABPP and this allows much higher resolution analysis of ABPP at the individual protein and protein isoform level, which should facilitate an even more effective use of activity probes.

### Host cell enzyme activity

This study marks the first use of these three classes of activity probes in Archaea. Two of the enzyme classes, serine hydrolase, and phosphatase, were expected to be present in *S. solfataricus* based on amino acid homology. Caspase-like activity had been suggested but never demonstrated. All three probes reacted with discrete protein spots and performed well using standard 2D gel running conditions (Figure 6). The benefit to this work was multi-fold. First, we have shown that standard activity probes can be effective with proteins from extremophiles, activity changes in specific biological pathways involved with viral infection could be targeted, and the identification of proteins that were targeted can be used to assist in gene annotation and establish cross-reactivity with other enzyme classes. When ABPP probes are coupled with 2D gel-based proteomics, enzyme cross-reactivity is actually an advantage. Up to a point, the more cross-reactivity a probe has, the wider the window of biological change that can be assessed. Specific to this work, phosphatases are clearly a part of the signaling during STIV infection, a number of proteins have cysteine residues with a nucleophilicity and selectivity similar to caspases, and ABPP expanded the accessible dynamic range in our experiments. In the big picture, these findings demonstrate the practicality and versatility of semi-directed ABPP to complement other omics methods and accelerate the discovery of altered protein activities.

We are just beginning to scratch the surface and see into the world of archaeal viruses and have glimpses of how they interact with their hosts. As these studies progress, the list of commonalities and differences between the three domains of life will surely continue to grow as will the potential for cross fertilization between mechanistic studies of Archaea, Eukarya, and Bacteria. The relatively small size of the *Sulfolobus* genome, the mix of eukaryotic and bacterial features, and the evolutionary relationship of STIV with viruses in all three domains makes this an ideal system to investigate viral host interactions.

## Conflict of Interest Statement

The authors declare that the research was conducted in the absence of any other commercial or financial relationships that could be construed as a potential conflict of interest. E.A. Dratz and P.A. Grieco are principles in Zdye LLC that has been developing zwitterionic dyes for proteomic analysis.

## Supplementary Material

The Supplementary Material for this article can be found online at http://www.frontiersin.org/Virology/10.3389/fmicb.2012.00411/abstract

Supplementary Table S1 **Regulated *S. solfataricus* (P2) proteins**.

Supplementary Table S2 **Sulfolobus pathways**.

Supplementary Table S3 **Identified proteins from phospho-stained spots**.

Supplementary Table S4 **Identifed ABPP labeled proteins**.

## Abbreviations

ABPP, activity-based protein profiling; LCMS, liquid chromatography-mass spectrometry; STIV, *Sulfolobus* turreted icosahedral virus.
